# Rebastinib attenuates liver injury following cecal ligation and puncture in male mice

**DOI:** 10.25122/jml-2023-0089

**Published:** 2023-11

**Authors:** Aula Zaini, Hayder Edrees Jawad, Dhefaf Hameed AL-Mudhafar, Najah Rayish Hadi

**Affiliations:** 1 Department of Pharmacology and Therapeutics, Faculty of Medicine, University of Kufa, Najaf, Iraq; 2 Middle Euphrates Cancer Center, Faculty of Medicine, University of Kufa, Najaf, Iraq

**Keywords:** Sepsis, inflammation, liver injury, Tie2 receptor, Rebastinib-treated group

## Abstract

Sepsis remains a public health issue with high morbidity and mortality. Liver injury due to sepsis is associated with poor outcomes and an increased risk of death among septic patients. Rebastinib is a small molecule inhibiting the Tie2 receptor and vascular endothelial growth factor receptor-2 (VEGFR2). The current study aimed to reveal the potential protective impact of Rebastinib against sepsis-induced liver injury. Twenty-four adult male mice were allocated into four groups (six per group) as follows: Sham group was exposed to anesthesia and laparotomy with no cecal ligation and puncture procedure (CLP); CLP group was subjected CLP procedure; vehicle-treated group was pretreated with vehicle (oral route) one hour prior to CLP procedure; Rebastinib group was pretreated with oral Rebastinib one hour before induction of CLP. Collected blood was used to measure the serum levels of AST, ALT, and angiopoietin 2. Homogenized liver tissues were used to investigate IL-6, TNF-α, ICAM-1, MIF, VEGF, F2-isoprostanes, and caspase-11 levels. Histological examination was used to determine the severity of liver damage. Compared to the sham group, mice subjected to CLP had high levels of these biomarkers with a high degree of liver injury. In contrast, Rebastinib markedly reduced these levels and mitigated the liver damage. Rebastinib may be a hepatoprotective agent against sepsis-associated liver injury.

## INTRODUCTION

Sepsis, a life-threatening condition marked by the host's excessive inflammatory response to an infection, significantly increases the risks of morbidity and mortality [1, 2]. Annually, the global incidence of sepsis is rising, with approximately eighteen million cases diagnosed, exerting substantial pressure on healthcare systems [3]. Despite intensive education and advanced therapeutic strategies, sepsis leads to over 10,000 deaths annually due to organ dysfunction [3, 4]. The pathogenesis of sepsis involves complex mechanisms, including inflammatory biomarkers, immune system responses, and coagulation processes [5]. The liver, a critical organ, plays a pivotal role in the progression of sepsis, and improved liver function has been linked to reduced morbidity and mortality in septic patients [6-8].

Systemic inflammation in sepsis triggers various molecular signaling pathways, including the angiopoietin-Tie2 axis, essential for endothelial cell regulation [9]. Rebastinib, a potent orally administered small molecule, acts as an inhibitor of multiple kinases, including Tie2 and vascular endothelial growth factor receptor 2 (VEGFR2) [10]. Recent preclinical studies in mouse models of metastasic carcinoma and pancreatic neuroendocrine tumors revealed that Rebastinib inhibits tumor growth, angiogenesis, and metastasis and increases the survival rate of mice treated with paclitaxel even after resection of the primary tumor [10]. So far, very little attention has been paid to the effect of Rebastinib on sepsis-induced liver injury. Thus, this study aimed to investigate the hepatoprotective potential of Rebastinib against liver injury caused by sepsis.

## MATERIAL AND METHODS

### Chemicals and ELISA kits

Rebastinib, SBE-β-CD (sulfobutylether-βcyclodextrin derivative used as excipient), and a protease inhibitor cocktail were procured from Med Chem Express. Angiopoietin 2 (Ang2), interleukin 6 (IL-6), tumor necrosis factor-alpha (TNF-α), macrophage inhibitory factor (MIF), caspase 11, F2-isoprostanes, intercellular adhesion molecule 1 (ICAM-1), and VEGF ELISA kits were obtained from Bioassay Technology Laboratory. Liver function tests, alanine aminotransferase (ALT), and aspartate aminotransferase (AST) from Roche Diagnostics. Ketamine vial (100 mg/ml, 50 ml) was obtained from Alfasan, and xylazine vial (20mg/ml, 25l) from Arendonk.

### Animal grouping

All experimental procedures were conducted at the Department of Pharmacology and Therapeutics, Cancer Research Unit, Faculty of Medicine, University of Kufa, Najaf, Iraq. Animals were housed at the animal care center of the Faculty of Science, University of Kufa, Iraq. Four groups of male mice (six per group), weighing 25-35 g and aged 8-12 weeks, were divided as follows:

The sham group underwent only a midline incision without cecal ligation and puncture (CLP). The CLP group underwent laparotomy followed by CLP. The vehicle-treated mice were given the vehicle of Rebastinib that was prepared as 10% dimethylsulfoxide (DMSO) + 90 % (20 % SBE-β-CD in saline) orally one hour prior to CLP. The Rebastinib-treated group received 100 mg/kg of Rebastinib orally one hour before CLP.

### CLP procedure

The CLP model was performed as previously described [8, 11]. Mice were anesthetized with ketamine (100 mg/kg) and xylazine (10 mg/kg) via intraperitoneal (i.p) route. The abdomen was disinfected, and a midline incision was made. The cecum was exposed and ligated below the ileocecal valve and punctured twice with a 22-gauge needle. The cecum was returned to its original position, and the abdomen was sutured with 6.0 stitches. Mice were returned to their cages with free access to food and water for 24 hours.

### Blood sampling

Twenty-four hours post-CLP, mice were euthanized, and approximately 1.5 ml of blood was collected from each mouse. The blood was allowed to clot for 20 minutes and then centrifuged at 2,000-3,000 rpm for 20 minutes. The serum was used to measure Ang2, ALT, and AST levels.

### ELISA method for liver tissue biomarkers

Liver samples from each mouse were excised, washed with cold PBS, and weighed. A PBS solution containing a 1% protease inhibitor cocktail and 1% Triton X-100 (1:9 W/V) was added to the liver samples, which were then homogenized using an ultrasonic processor. To prepare the supernatants, the processed liver samples were centrifuged at 12,000 rpm for 15 min at 4^o^C. The supernatants were used to examine the levels of IL-6, TNF-α, MIF, ICAM-1, F2-isoprostanes, VEGF, and caspase 11.

### Histological examination

Liver specimens were fixed in 10% formalin, embedded in paraffin, and sectioned into 5-micrometer slices. Liver sections were then stained with hematoxylin and eosin (H & E) and examined under a light microscope by a pathologist who was blind to the study groups. The degree of liver damage was scored from 0 to 3 based on histopathological changes (vacuoles, ballooning, pyknosis, apoptosis, necrosis), as detailed in Table 1.

**Table 1:** Liver injury scores

Scores	Levels of liver damage
0	Normal
1	Mild
2	Moderate
3	Severe

### Statistical analysis

The data collected from this study were analyzed and visualized using GraphPad Prism 7 software. Results are expressed as mean ± standard error of the mean (SEM), providing a clear and standardized data representation. Statistical differences between groups were determined using analysis of variance (ANOVA), followed by Bonferroni's correction for Type I error to adjust for multiple comparisons. For the non-parametric histological scores, the Kruskal-Wallis test was employed. A p-value of ≤0.05 was considered statistically significant.

## RESULTS

### Liver function

**Figure 1:**
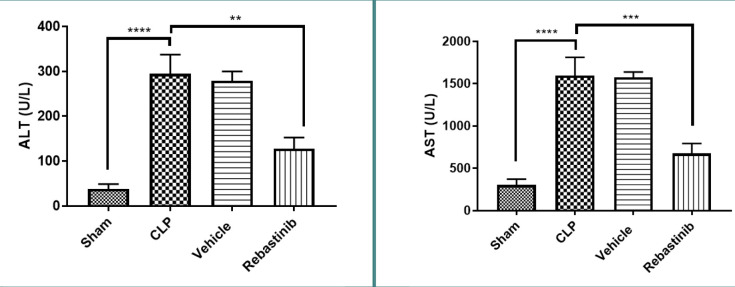
Impact of Rebastinib on serum ALT and AST levels post-CLP Serum ALT and AST levels across groups are shown as mean±SEM (n=6). One-way ANOVA and Bonferroni test; significance levels: **p≤0.01, ***p≤0.001, ****p≤0.0001.

Serum levels of AST and ALT were significantly higher in the CLP group compared to the sham group (p≤0.05) (Figure 1). Rebastinib treatment significantly reduced these levels, suggesting improved liver function (p≤0.05, Figure 1).

### Inflammatory biomarkers in liver tissue

**Figure 2:**
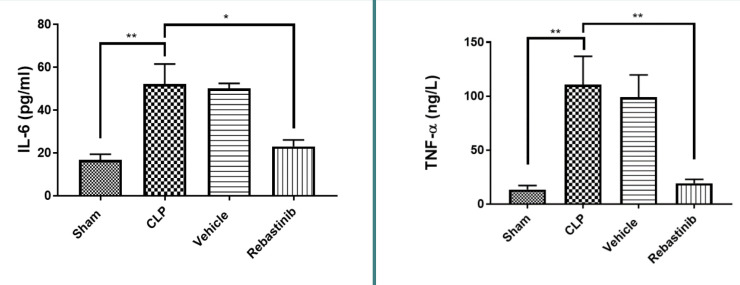
Effect of Rebastinib on IL-6 and TNF-α levels in the liver Tissue levels of IL-6 and TNF-α across groups, shown as mean±SEM (n=6). One-way ANOVA and Bonferroni test; *p≤0.05, **p≤0.01.

IL-6 and TNF-α levels in liver tissues were significantly elevated in the CLP group compared to the sham group (p-value≤0.05, Figure 2). Rebastinib treatment significantly reduced these levels (p≤0.05, Figure 2).

### Oxidative stress and apoptosis biomarkers

**Figure 3:**
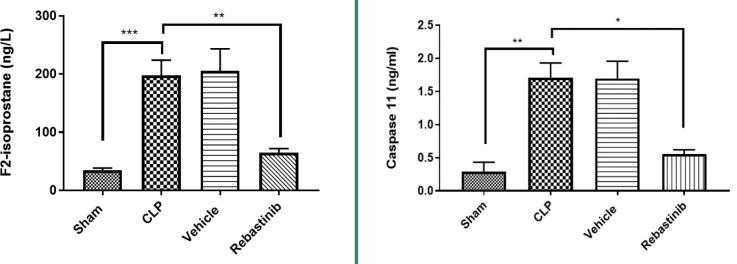
Effect of Rebastinib on F2-isoprostane and caspase 11 levels in the liver Tissue levels of F2-isoprostane and caspase 11 across groups, shown as mean±SEM (n=6). One-way ANOVA followed by Bonferroni test; *p≤0.05, **p≤0.01, ***p≤0.001.

F2-isoprostane and caspase 11 levels in liver tissues were significantly higher in the CLP group than in the sham group (p-value≤0.05, Figure 3). Rebastinib treatment resulted in lower levels of these biomarkers compared to the CLP group (p-value≤0.05, Figure 3).

### Effect of Rebastinib on MIF and ICAM-1

**Figure 4:**
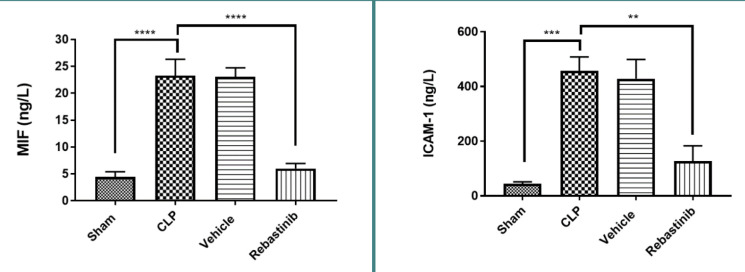
Effect of Rebastinib on tissue levels of MIF and ICAM-1 Tissue levels of MIF and ICAM-across groups are shown as mean±SEM (n=6). One-way ANOVA followed by Bonferroni test; **p≤0.01, ***p≤0.001, ****p≤0.0001.

Levels of MIF and ICAM-1 in the liver were dramatically higher in the CLP group than in the sham group (p-value ≤0.05, Figure 4). These levels were significantly decreased in mice treated with Rebastinib (p-value≤0.05, Figure 4).

### Angiogenic factors

**Figure 5:**
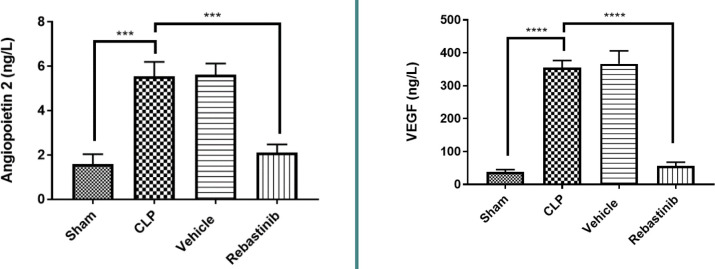
Effect of Rebastinib on angiopoietin 2 and VEGF levels Serum levels of Ang2 and tissue levels of VEGF across groups are shown as mean±SEM (n=6). One-way ANOVA followed by Bonferroni test; ***p≤0.001, ****p≤0.0001.

Serum levels of Ang2 and tissue levels of VEGF were significantly higher in the CLP group compared to the sham group (p-value≤0.05, Figure 5). Rebastinib treatment significantly reduced these levels, indicating an antiangiogenic effect (p-value≤0.05, Figure 5).

**Figure 6:**
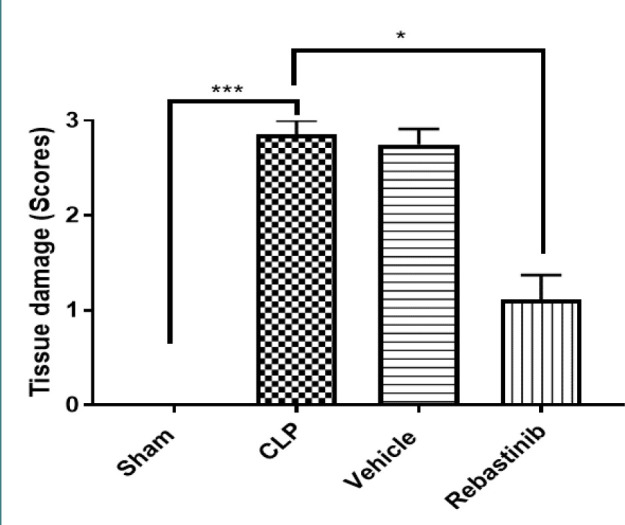
Effect of Rebastinib on liver injury Liver injury scores across groups are shown as mean±SEM (n=6). Kruskal Wallis test and Bonferroni test; *p≤0.05, ***p≤0.001.

### Histological analysis of liver damage

**Figure 7:**
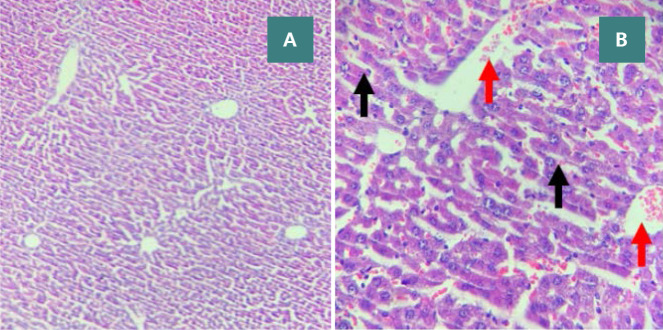
Sham group: normal liver histology Liver tissue sections displaying a central vein (red arrow) and normal hepatocytes (black arrows), stained with H&E. Magnifications: A - 100x, B - 400x.

**Figure 8:**
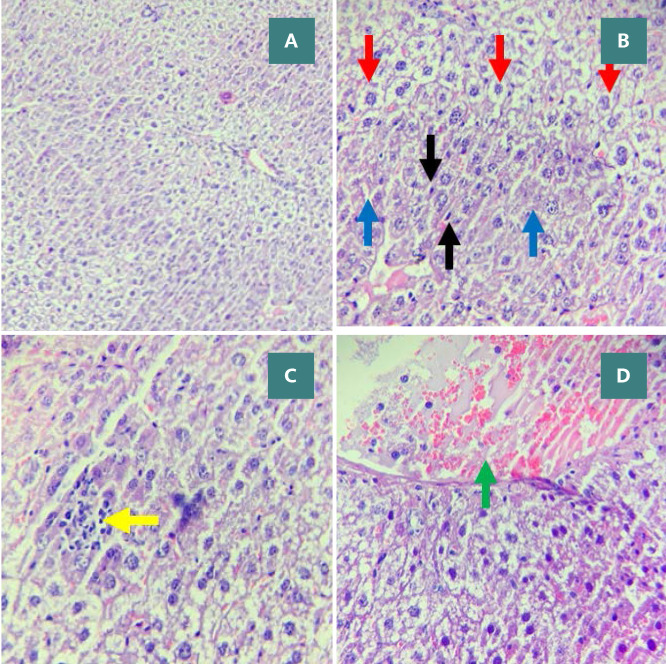
CLP group: severe liver damage (score 3) Liver sections show cytoplasmic eosinophilia, apoptotic cells with chromatin condensation, pyknotic nuclei (black arrows), necrotic cells with nuclear fading (red arrows), steatosis (blue arrows), focal mild inflammation (yellow arrow), and vascular congestion (green arrow). Stained with H&E. Magnifications: A - 100x, B/C/D - 400x.

**Figure 9:**
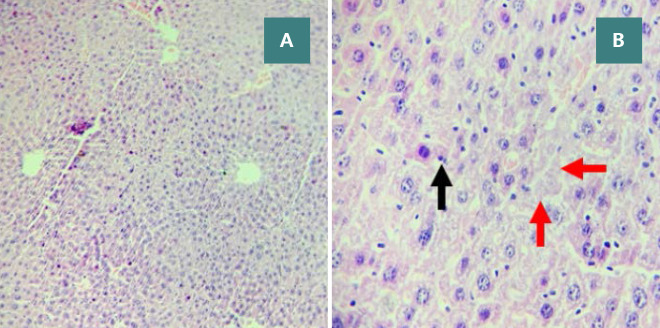
Vehicle group: severe liver damage (Score 3) Liver sections characterized by cytoplasmic eosinophilia, apoptotic cells with chromatin condensation, and pyknotic nuclei (black arrows). Necrotic cells with nuclear fading (red arrows). H&E. Magnifications: A - 100x, B - 400x.

**Figure 10:**
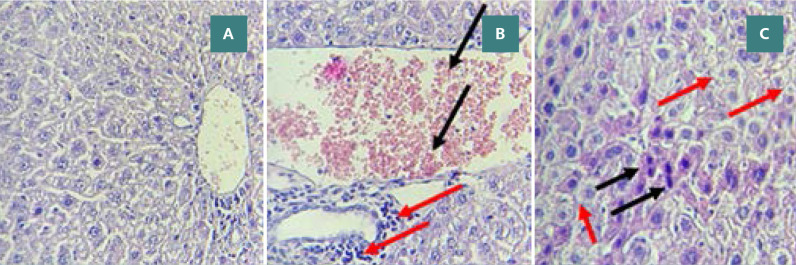
Effect of Rebastinib on liver tissue post-CLP A: Mild liver damage (Score 1) with cytoplasmic vacuoles (400x magnification). B: Vascular congestion with erythrocyte stasis (black arrows) and mild inflammation (red arrows) (400x magnification). C: Moderate liver damage (Score 2) featuring cytoplasmic eosinophilia, apoptotic cells with chromatin condensation, pyknotic nuclei (black arrows), and cytoplasmic vacuoles (red arrows) (400x magnification)

Histological analysis showed significant liver damage in mice subjected to CLP, as evidenced by high damage scores and liver sections showing apoptosis and necrosis, compared to the sham group (p-value≤0.05, Figures 6, 7A-B, and 8A-D). There were no significant changes between the CLP and vehicle-treated groups (Figures 6 and 9A-B). However, compared to the CLP group, treatment with Rebastinib ameliorated the structural features of liver tissues and reduced the severity of liver damage (Figures 6 and 10A-C).

## DISCUSSION

This study revealed the beneficial role of Rebastinib in ameliorating liver injury in septic mice via modulating the Tie2/angiopoietin signaling pathway, which is a crucial mediator in sepsis. Sepsis, characterized by an overwhelming host response to infection, significantly impacts multiple organs, including the liver, kidneys, and heart. Liver damage in sepsis is a key factor in patient mortality, necessitating prompt and effective treatment strategies [12, 13]. Excessive inflammatory response and oxidative stress play pivotal roles in the pathogenesis of liver injury, making them key targets for therapeutic intervention [14]. Rebastinib is a selective inhibitor of the Tie2 receptor, which is expressed on vascular endothelial cells and tumor-associated macrophages that express Tie2 [10, 15]. In tumors, this small molecule antagonizes the Tie2 receptor, resulting in a variety of effects on tumor cells, such as the reduction in growth, dissemination, and metastasis [10]. Rebastinib has obtained extensive attention in treating some types of cancers exploiting the angiopoietin/Tie2 signaling axis. However, its potential in treating sepsis-induced organ damage remains underexplored. The present study revealed that treatment with Rebastinib reduced the levels of inflammatory cytokines, such as TNF-α, MIF, and IL-6, suggesting that it could have an anti-inflammatory impact. This is the first study to highlight the potential of this drug in decreasing inflammatory readouts. In addition, Rebastinib treatment markedly decreased F2-isoprostane levels compared to the CLP group. No previous reports have examined the role of Rebastinib in models of sepsis and particularly its impact on levels of F2-isoprostane. This marker is extensively studied as one indicator of oxidative stress lipid peroxidation as it is characterized by high stability in tissues, urine, and blood; thereby, it is a critical factor in sepsis [16]. It has been reported that increased levels of F2-isoprostane contribute to liver and kidney failure [17]. Our study showed that treatment with Rebastinib significantly reduced ICAM-1 levels compared to the CLP group. Until now, no studies have investigated the potential effect of Rebastinib on ICAM-1 levels following CLP. Sepsis leads to widespread disruption of cellular components and tissues, including the endothelium, which is highly responsive to inflammatory stimuli. A critical factor in this process is ICAM-1, a molecule that increases during sepsis alongside other pro-inflammatory cytokines. This elevation in ICAM-1 contributes to the recruitment of leukocytes and a state of coagulation, ultimately leading to apoptosis and necrosis of endothelial cells [18]. Rebastinib, therefore, could be a protective agent against sepsis-provoked liver injury via its influence on ICAM-1 levels. This study also revealed that treatment with Rebastinib significantly reduced caspase 11, suggesting its antiapoptotic effect. No previous reports illustrated the effect of Rebastinib on levels of caspase 11, and further research regarding the role of Rebastinib would be worthwhile.

Furthermore, Rebastinib treatment significantly reduced VEGF levels compared to the CLP group, suggesting its potential antiangiogenic effects. To our knowledge, no reports have specifically addressed the impact of Rebastinib on VEGF levels. VEGF is a critical regulator in the dysregulation of endothelial cells, acting as a compensatory response by the host to counteract the unwanted inflammatory responses induced by sepsis [19]. VEGF increases during sepsis, leading to vascular leakage and augmentation of the inflammatory response [20]. Therefore, reduced levels of VEGF by Rebastinib could have a protective impact against sepsis. In addition, Rebastinib significantly decreased serum levels of angiopoietin 2 compared to the CLP group, being the first study to investigate the impact of Rebastinib on serum levels of angiopoietin 2. The angiopoietin 2/Tie2 signaling axis plays a vital role in endothelial cells, influencing vascular stability and angiogenesis [21, 22]. In sepsis, there is an imbalance between the angiopoietin 1 and angiopoietin 2 ratio, with a relative increase in angiopoietin 2 levels [23]. This imbalance contributes to endothelial destabilization and inflammation, which are further exacerbated by VEGF, thus initiating organ damage following sepsis. [24]. Rebastinib, a Tie2 inhibitor, can interact and modulate the Tie2 signaling pathway, which could be a key player in controlling sepsis-associated liver injury. Finally, ALT and AST indicators were lower in the Rebastinib-treated group compared to the CLP. This suggests that Rebastinib ameliorated liver function. In addition, it positively affected the morphology of liver tissue. The liver tissues in the Rebastinib-treated group showed characteristics closer to those of the sham group, which did not undergo the CLP procedure.

## CONCLUSION

The present study revealed that Rebastinib had protective effects on liver injury induced by CLP via its anti-inflammatory, antiangiogenic, and anti-apoptotic properties.
